# Genetically edited human placental organoids cast new light on the role of ACE2

**DOI:** 10.1038/s41419-025-07400-x

**Published:** 2025-02-07

**Authors:** Anya L. Arthurs, Bianca Dietrich, Martin Knöfler, Caleb J. Lushington, Paul Q. Thomas, Fatwa Adikusuma, Jessica M. Williamson, Susan Babikha, Tyla Damhuis, Tanja Jankovic-Karasoulos, Melanie D. Smith, Kirsty G. Pringle, Claire T. Roberts

**Affiliations:** 1 Flinders University, College of Medicine and Public Health, Flinders Health and Medical Research Institute, Adelaide, SA Australia; 2 Placental Development Group, Medical University of Vienna, Vienna, Austria; 3 School of Biomedicine and Robinson Research Institute, University of Adelaide, Adelaide, SA Australia; 4 Genome Editing Program, South Australian Health and Medical Research Institute (SAHMRI), Adelaide, SA Australia; 5 South Australian Genome Editing (SAGE), South Australian Health and Medical Research Institute (SAHMRI), Adelaide, SA Australia; 6 School of Biomedical Sciences and Pharmacy, College of Health, Medicine and Wellbeing, University of Newcastle, Callaghan, NSW Australia; 7 Mothers and Babies Research Program, Hunter Medical Research Institute, New Lambton Heights, Newcastle, NSW Australia

**Keywords:** Genetics research, Developmental biology, Biochemistry

## Abstract

ACE2 expression is altered in pregnancy disorders and *ACE2* gene variants are associated with several major pregnancy complications including small-for-gestational-age, fetal growth restriction and preeclampsia. This study utilised gene-editing to generate both *ACE2* knockout and *ACE2* rs2074192 placental organoids, facilitating mechanistic studies into the role of *ACE2* in placental development, and the effect of fetal carriage of *ACE2* rs2074192 CC, CT and TT genotypes. Parameters of cell and organoid growth were measured, together with qPCR, Western Blotting, and ELISA assessments, in all groups from both organoid models. Here, we report that *ACE2* knockout results in delayed placental cell growth and increased cell death. *ACE2* knockout organoids had lower ACE protein expression, reduced organoid diameters and asymmetrical growth. Placental organoids with the ACE2 rs2074192 TT genotype had significantly higher expression of *ACE2* mRNA and ACE2 protein with elevated ACE2:ACE expression ratio and no change in ACE protein. Despite increased expression of ACE2 protein, ACE2 enzyme activity was significantly decreased in ACE2 rs2074192 TT placental organoids. TT organoids also had reduced diameters and asymmetrical growth. Our research provides a new molecular understanding of the role of ACE2 in placental development, with potential implications for pregnancy in the carriage of the *ACE2* rs2074192 gene variant.

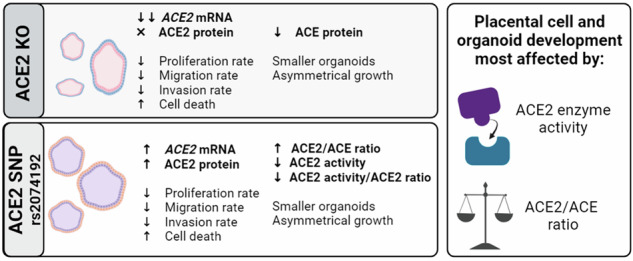

## Introduction

Understanding the molecular mechanisms underlying pregnancy and foetal development is crucial for improving maternal and neonatal health outcomes. Despite significant advances in obstetric care, complications such as small-for-gestational-age, foetal growth restriction and preeclampsia continue to pose substantial risks. Recent studies have highlighted the pivotal role of placental function in these conditions, with literature suggesting that variations in placental angiotensin-converting enzyme 2 (*ACE2*) gene expression may be a key factor. However, there remains a gap in our comprehensive understanding of how *ACE2* genetic variations and molecular pathways contribute to these pregnancy disorders.

**Fig. 1:**
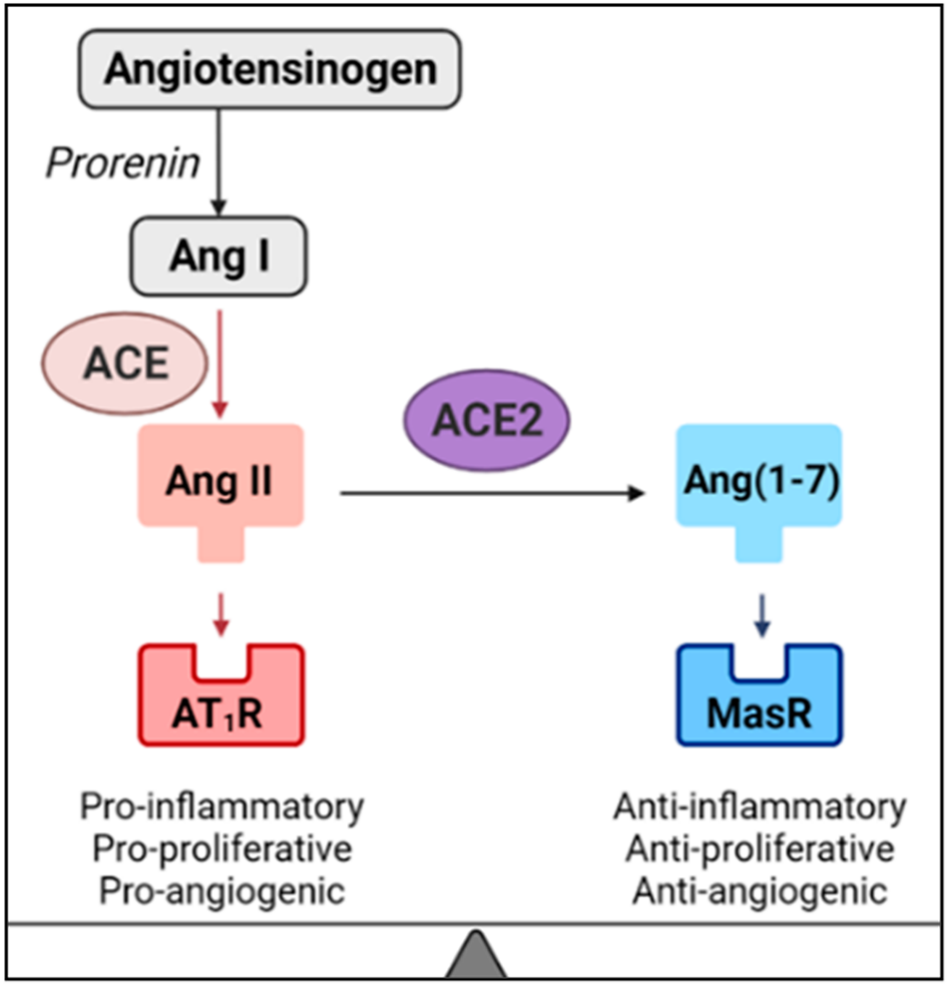
The ACE- and ACE2-mediated opposing arms of the renin-angiotensin system.

Interest in previously little-known ACE2 has escalated with the COVID-19 pandemic and the knowledge that ACE2 is the SARS-CoV-2 receptor. However, the role of ACE2 in the placenta remains understudied, despite its evident importance in pregnancy. Pregnant women have higher circulating levels of ACE2 compared to non-pregnant women [1]. ACE2 abundance is altered in pregnancy disorders, including preeclampsia, small for gestational age (SGA) and foetal growth restriction [1–7], and *ACE2* gene variants are associated with several major pregnancy complications [1, 5, 8]. Genetic factors account for two-thirds of the phenotypic variation in circulating ACE2 [9]; specifically, *ACE2* single nucleotide polymorphisms (SNPs) and haplotypes are associated with the altered abundance of Angiotensin II (Ang II) and Angiotensin 1–7 (Ang 1–7), which are the peptides produced by angiotensin-converting enzyme (ACE) and ACE2 catalytic activity, respectively [10] (Fig. 1).

The circulating renin-angiotensin system is a critical regulator of blood volume and systemic vascular resistance. However, in addition to the circulatory system, local tissue-specific renin-angiotensin systems exist, including within the placenta. The pro-inflammatory, pro-proliferative arm of the placental renin-angiotensin system (shown in red in Fig. 1), mediated by ACE, promotes cell and tissue growth. This is opposed by an anti-inflammatory, anti-proliferative arm (shown in blue in Fig. 1), mediated by ACE2 [3]. Both enzymes catalyse the formation of peptides responsible for binding to separate angiotensin receptors, thus initiating their own signalling cascades (including MAPK/ERK and p85-PI3K) which contribute to tissue growth and vascularisation [11, 12]. ACE and ACE2 maintain a finely tuned balance within the renin-angiotensin system to optimise placental growth and function throughout a healthy pregnancy. However, the expression and activity of ACE and ACE2 enzymes, both systemically [1, 2] and in the placenta [3–7], are disturbed in many pregnancy complications associated with renin-angiotensin system dysfunction.

Adequate ACE2 expression and activity, in both the mother and placenta, is necessary for a healthy pregnancy. In *ACE2*^−/−^ (*ACE2* knockout) mouse models, *ACE2* knockout dams have higher systolic blood pressure [2, 4], decreased plasma Ang 1–7 [2], and placentae have increased levels of [2], and sensitivity to [4], Ang II. This results in alterations to the balance of ACE and ACE2 activity in the renin-angiotensin system. Furthermore, pups from *ACE2* knockout dams have reduced weight [2, 4] and length [2], with lower pup-to-placenta weight ratios, indicating compromised placental function. Clearly, ACE2 is essential for healthy pregnancy but dysregulation of ACE2 levels and activity, as well as ACE:ACE2 balance, is pathogenic [4, 13]. However, whilst murine research is informative, an *ACE2* knockout model of the human placenta has never been established.

As previously mentioned, *ACE2* gene variants (including single nucleotide polymorphisms [SNPs]) alter the expression of renin-angiotensin system components. An SNP is a single base substitution at a specific chromosome location. Many SNPs in the *ACE2* gene have been associated with disease states [5, 8, 14–16]. In particular, *ACE2* rs2074192 is associated with an increased incidence of diseases characterised by renin-angiotensin system dysregulation [14–17], including increased susceptibility to COVID-19 and increased disease severity [15, 16]. Importantly for pregnancy, SGA, a condition where neonatal birthweight is lower than expected for their gestational age, has been associated with *ACE2* rs2074192 (C to T polymorphism) which, when carried by the foetus, increases the risk of SGA by 23-fold [5]. The placenta, a product of conception, is genetically identical to the foetus. As such, a foetal SNP will also be present in the placenta. Despite strong associations between *ACE2* rs2074192 frequency and disease state, and the estimated frequency of at least one copy of this SNP in one-third of the global population [18], research is mostly limited to association studies but not its mechanisms of action.

In this study, we utilised gene-editing to generate *ACE2* knockout placental organoids and *ACE2* rs2074192 placental organoids, creating the first induced SNP gene-edited organoids in the human placenta. We investigated the role of *ACE2* in placental development by profiling the expression of *ACE2* mRNA and protein, ACE protein, ACE2 enzyme activity and parameters of cell and organoid growth in both placental organoid models. Elucidating the role of *ACE2* in the placenta will allow inferences to be made regarding its overall contribution to pregnancy health, as well as its possible role in other physiological and pathological states that involve the renin-angiotensin system.

## Materials and methods

Trophoblast stem cells (TSCs) were isolated from *n* = 3 patients and grown to confluence. At this point, TSCs from each patient were separated into two groups:

1. *ACE2*^−^*/*^−^ (^*ACE2*^ knockout—hereafter referred to as *ACE2*^*+/+*^*, ACE2*^*+/*^^−^ or *ACE2* KO).
2. *ACE2* SNP (rs2074192—hereafter referred to by genotype, i.e. CC, CT, or TT).

**Fig. 2:**
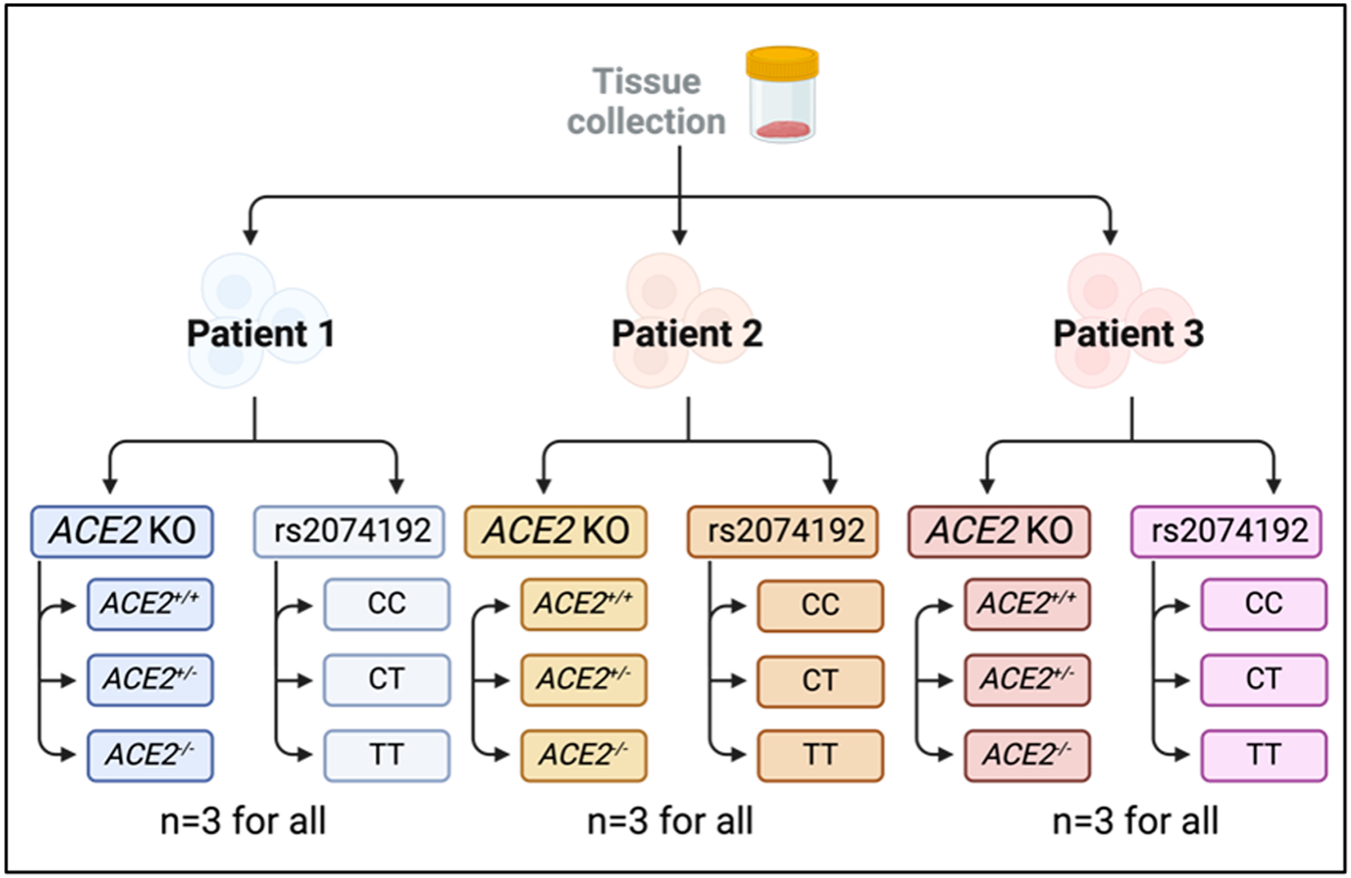
Method describing gene-editing to generate different TSC groups. Early gestation placenta tissue was collected from *n* = 3 patients. For each tissue sample, trophoblast stem cells (TSCs) were isolated and cultured prior to separating into two groups: **(1)** *ACE2* KO and **(2)** rs2074192. Within group **(1)**, cells were transfected to achieve genotypes of two copies of the ACE2 gene (ACE2^+/+^), one copy (ACE2^+/-^) or zero copies (ACE2^-/-^). Within group **(2)**, cells were transfected to achieve genotypes of two dominant alleles (CC), one dominant and one recessive allele (CT) or two recessive alleles (rs2074192 SNP; TT). Once successfully transfected, cells were used to create organoids. All experiments performed in technical triplicate.

The generation of gene-edited organoids from each patient was replicated three times, thus a final *n* = 9 independently generated organoids for each genotype group (see Fig. 2).

As *ACE2* is on the X chromosome, only placentae from female fetuses were included to allow analysis of a heterozygous group (*ACE2*^*+/*^^−^).

### Tissue collection

First trimester (6–7 weeks’ gestation) human placentae were obtained with informed consent from women undergoing elective termination of pregnancy at the Pregnancy Advisory Centre in the Queen Elizabeth Hospital, in Woodville, South Australia. Ethics approval was obtained from the Central Adelaide Local Health Network Human Research Ethics Committee, HREC/16/TQEH/33, Q20160305. Placentae were collected within minutes of termination upon which villus tissue was washed with Hanks’ Balanced Salt Solution (HBSS; Gibco, Sigma-Aldrich) and transported to the laboratory on ice.

### Genotyping for foetal sex

Placentae from *n* = 5 patients were genotyped for foetal sex high-resolution melt curve analysis of the gene that encodes amelogenin. Amelogenin is found on both the X (AMELX) and Y (AMELY) chromosomes, the X allele features a 3 bp deletion in exon 3 allowing the identification of samples with only X chromosomes (female) or X and Y chromosomes (male). Forward primer 5ʹ-CCCTGGGCTCTGTAAAGAATAGTG-3ʹ, reverse primer 5ʹ-ATCAGAGCTTAAACTGGGAAGCTG-3. qPCR was performed using SSOFast EvaGreen Supermix (Bio-Rad, CA), primers at 250 nM final concentration, 5 ng of DNA per reaction, on a Bio-Rad CFX384 Real-Time PCR System. Cycling conditions: initial denaturation 98 °C 30 s, 40 cycles of 98 °C for 5 s and 60 °C for 5 s. High-resolution melt curve analysis was performed from 65 °C to 85 °C with a 0.2 °C increment every 10 s. The melt curve between 65 °C and 70 °C was analysed using Bio-Rad Precision melt software (Bio-Rad, CA) to identify sex genotypes. *N* = 3 of these placentae, determined to be from female fetuses, were selected for use in this study.

### Isolation and cultivation of TSCs

TSCs were isolated from first-trimester placentae according to Dietrich et al. [19] Briefly, first-trimester villus tissue was washed in HBSS (4 °C) prior to manually isolating villus structures for further processing. Villi in HBSS were centrifuged (1000 rpm, 1 min) prior to three consecutive enzymatic digest steps and density gradient centrifugation as per Haider et al. [20]. After careful collection of cells between the 35% and 50% Percoll layers, cells were thoroughly washed with HBSS before seeding in fibronectin-coated culture plates using a culture medium to promote stemness. As per Dietrich et al. [19], TSC medium contained DMEM/F12 (Gibco) supplemented with 1 × B-27 (Gibco), 1 × Insulin-Transferrin-Selenium-Ethanolamine (ITS-X; Gibco), 1 µM A83-01 (Tocris), 50 ng/mL recombinant human epidermal growth factor (rhEGF; R&D Systems), 2 µM CHIR99021 (Tocris) and 5 µM Y27632 (Santa Cruz).

### Generation of plasmids

To generate modified ACE2 organoid models, two distinct gene editing strategies were employed. Firstly, a modified pDG459 plasmid (Addgene #100901) was designed to induce a complete *ACE2* knockout (KO). This entailed the precise targeting of regions of approximately 100 base pairs upstream and downstream of the first and last *ACE2* exons, resulting in the excision of promoter, regulatory, and coding sequences. Secondly, a modified PEA1-Puro plasmid (Addgene #171991) was designed to introduce an intronic *ACE2* single nucleotide polymorphism (SNP), specifically rs2074192. The SNP was targeted using the PE3 strategy, which incorporated a downstream nicking single-guide RNA (sgRNA) positioned 72 base pairs from the primary cleavage site, adhering to the recommended range of 40–90 nucleotides [21]. Construct assembly was achieved through *BbsI*-mediated golden gate assembly of phosphorylated and annealed oligonucleotide guide sequences (Thermo Fisher), following optimised protocols for PEA1 or pDG459 assembly [22, 23]. Subsequent plasmid purification was performed using the QIAprep Spin Miniprep Kit (Qiagen), and validation of oligonucleotide insertion was carried out through Sanger sequencing (AGRF). Oligonucleotide sequences are listed in Table 1.

**Table 1:** Sequences of oligonucleotides used to generate plasmids for organoid models.

Oligonucleotide	Oligonucleotide sequence 5’–3’
ACE2 KO Guide Promoter Oligo Forward	caccgCTGTCATTTCAGAATAATGCT
ACE2 KO Guide Promoter Oligo Reverse	aaacAGCATTATTCTGAAATGACAGc
ACE2 KO Guide Terminator Oligo Forward	accgCCTGACAGCTCATCATGAGAgt
ACE2 KO Guide Terminator Oligo Reverse	tgaaacTCTCATGATGAGCTGTCAGG
ACE2 SNP PE Nick sgRNA Guide Oligo Forward	accgGTAGATGTACTCTGACAGAAgt
ACE2 SNP PE Nick sgRNA Guide Oligo Reverse	tgaaacTTCTGTCAGAGTACATCTAC
ACE2 SNP PE Spacer Guide Oligo Forward	caccTGTGGAAATGTATAAATGGT
ACE2 SNP PE Spacer Guide Oligo Reverse	aaacACCATTTATACATTTCCACA
ACE2 SNP PE Repair Template Guide Oligo Forward	gtgcCAAATGAATAAATaCCAACCATTTATACATTTCCA
ACE2 SNP PE Repair Template Guide Oligo Reverse	aaaaTGGAAATGTATAAATGGTTGGtATTTATTCATTTG

### TSC genetic modification

TSCs were transfected with *ACE2* KO and *ACE2* SNP plasmids using DNAfectin Plus Transfection Reagent (abm), according to the manufacturer’s instructions. Briefly, media was aspirated from the TSC culture wells prior to the addition of the DNA-transfection complex. Control wells received only the transfection reagent without DNA plasmid. After 6 h the transfection solution was removed, and a complete medium was added. After 24 h, puromycin (0.2 µg/mL) was added to the culture media to begin antibiotic selection of successfully transfected cells. Puromycin selection continued for 14 days of culture (until cells reached confluence). All TSC and organoid culture was conducted at 37 °C, 5% O_2_ (to mimic the *in utero* first-trimester placental oxygen tension).

### Assessment of time to confluence and cell death

Immediately after culture with puromycin, gene-edited TSCs were passaged using TrypLE Reagent (TrypLE, Gibco), according to manufacturer’s instructions, then replated at a density of ~20% (1:5 dilution) per well. Cells were then grown in the Incucyte® SX5 Live-Cell Analysis System (Sartorius) for 4 days (until confluence). Cell confluence percentage and cell death were assessed using Incucyte analysis software. Once cells reached confluence (up to 96 h), TSCs were removed from the Incucyte® and organoids were created.

### Proliferation and migration analysis

Proliferation analysis was performed as described in Arthurs et al. 2019 [24], with minor alterations; the incubation medium was the TSC medium as described earlier, and 5 × 10^4^ TSCs from passage two were plated in each well. Cell proliferation was analysed from 24 h post-plating to allow for cell equilibration and adherence to the culture plate.

For cell migration analysis, an xCELLigence CIM-Plate 16 (ACEA Biosciences Inc., San Diego, CA) was used containing two chambers. One hundred sixty microliters of TSC media, as previously described, with 10% FBS, was added to each well of the lower chamber. Fifty microliters of serum-free media was added to each well of the upper chamber, and the CIM-plate was equilibrated for 1 h at 37 °C, before a background measurement was taken using the RTCA software (Agilent, Santa Clara, CA, USA). A total of 3 × 10^4^ TSCs were plated in each well of the upper chamber before equilibrating for 30 min at room temperature. CIM plates were placed in the RTCA DP cradle and cell index readings were taken every 30 min for 72 h using the RTCA software (Agilent, Santa Clara, CA, USA).

### Organoid formation

Placental organoids were established as per Haider et al. [20], with minor modifications. Briefly, gene-edited TSCs were trypsinised (TrypLE, Gibco) and washed in ice-cold advanced Dulbecco’s Modified Eagle Medium (DMEM, Gibco). Cells were resuspended in organoid media: ice-cold advanced DMEM supplemented with 1 × B-27, 1 × ITS-X, 10 mM HEPES (Gibco), 2 mM glutamine (Gibco), 1 µM A8391, 100 ng/mL rhEGF and 3 µM CHIR99021. Vitrogel (ORGANOID-1, The Well BioScience) was added to reach a 2:1 Vitrogel-to-organoid media ratio. Seventy-five microliters of the resultant mixture were plated on a 6-well culture plate, forming a dome in the centre of the well. After incubation (37 °C, 2 h), 1.5 mL organoid media (37 °C) was added to each well. Media was changed every 5 days until organoids reached confluence, at which point they were harvested for RNA and protein experiments.

### Genomic DNA (gDNA) extraction

gDNA extraction protocol was adapted from Ghatak, et al. [25]. Prior to organoid generation, a subset of TSCs from each gene-edited patient sample was harvested and gDNA was extracted by incubating cells with phenol:chloroform:isoamyl alcohol solution (25:24:1). After centrifugation (10 min, 10,000 × *g*, 4° C), the upper aqueous layer was collected and transferred to a new tube. RNase A (0.1 mg; Lucigen) was added. Isopropanol (4 °C, 1:1 volume ratio) and sodium acetate (4 °C, 3 M, 1:10 volume ratio) were added to the mixture prior to precipitation (−20 °C, 1 h). The sample was then centrifuged (10,000 × *g*, 10 min, 4 °C) before the supernatant was decanted. The pellet was washed in 250 µL 75% EtOH prior to centrifugation (10,000 × *g*, 10 min, 4 °C). The supernatant was again decanted, and the pellet was left to air-dry, then resuspended in nuclease-free H_2_O (30 µL).

### Genotyping for *ACE2* rs2074192

Genotyping was conducted by the Australian Genome Research Facility (AGRF) using the Sequenom MassARRAY system, as per Zhou et al. [26].

### RNA extraction

Culture media was aspirated from organoid wells and organoids were gently washed with phosphate-buffered saline (PBS). Tissue was disrupted by homogenizing for 3.5 min at 30 Hz (TissueLyser, QIAGEN) in 600 μL Buffer RLT Plus (RNeasy Plus Mini Kit; QIAGEN, Victoria, Australia). Total RNA was extracted from the supernatant using TRIzol reagent as per the manufacturer’s instructions and as outlined by Rio et al. [27]. The purity and integrity of extracted RNA samples were determined using the NanoDrop Spectrophotometer (Thermo Fisher Scientific) and samples used had 260:280 and 260:230 nm ratios greater than 1.9.

### cDNA synthesis and quantitative polymerase chain reaction (qPCR)

As previously described [28], the synthesis of complementary DNA (cDNA) was conducted beginning with 1 μg of total RNA using the QuantiNova Reverse Transcription Kit (QIAGEN) according to the manufacturer’s protocol. qPCR was conducted with SYBR Green (QIAGEN) according to the manufacturer’s instructions, with YWHAZ (*YWHAZ*) and β-actin (*ACTINB*) as housekeeping genes (for primer sequences, see Table 2). Denaturation was performed at 95 °C for 10 s, annealing at 53 °C for 45 s, and extension at 72 °C for 30 s, for a total of 50 cycles. qPCR results were analysed using the 2^−ΔΔCT^ method [29].

**Table 2:** Sequences for qPCR primers.

Gene	Primer sequence (forward)	Primer sequence (reverse)
*ACE2*	CAGGGAACAGGTAGAGGACATT	CAGAGGGTGAACATACAGTTGG
*ACTINB*	CACCATTGGCAATGAGCGGTTC	AGGTCTTTGCGGATGTCCACGT
*YWHAZ*	ACCGTTACTTGGCTGAGGTTGC	CCCAGTCTGATAGGATGTGTTGG

### Protein extraction

Total protein was extracted from gene-edited placental organoids using a radioimmunoprecipitation assay (RIPA) lysis and extraction buffer. Organoids were harvested from culture wells and each sample was placed in 400 µL ice-cold RIPA buffer with added Complete Mini Protease Inhibitor Cocktail tablet (Roche Diagnostics Australia) and phenylmethylsulfonyl fluoride (0.4 pmol). Samples were incubated (10 min, 4 °C), vortexed, and centrifuged (16,000 ×g, 10 min, 4 °C) and supernatants were collected. Protein concentration was measured via a Bradford Assay using the SpectraMax iD5 (Molecular Devices) at 595 nm absorbance.

### SDS-PAGE

As previously described [28], samples were denatured and reduced in Laemmli 4x buffer (GTX16355, GeneTex) and 8% 2-mercaptoethanol (5 min, 95 °C). Samples were loaded onto either a 4–20% Mini-PROTEAN^®^ TGX Stain-Free™ Protein Gel (4568094, Bio-Rad) or 4–20% Mini-PROTEAN^®^ TGX™ Precast Protein Gel (4561094, Bio-Rad), along with a molecular weight marker (GTX49384, GeneTex). SDS-PAGE was performed (1 h, 100 V) in 1× Running buffer (pH 8.3) using a Mini-PROTEAN Tetra Vertical Electrophoresis Cell (Bio-Rad). Stain-free protein gels were checked for complete protein separation using the ChemiDoc Touch Imaging System (Bio-Rad).

### Transfer

Protein was transferred to a PVDF membrane over 16 h (27 V, 4 °C) in transfer buffer (25 mM Trizma Base, 190 mM glycine, 20% methanol; pH 8.3) using the Criterion™ blotter (Bio-Rad). Successful transfer of protein was checked by Ponceau S staining of the membrane, as well as either Coomassie Blue staining (for the TGX Precast Protein Gel) or the ChemiDoc Touch Imaging System (Bio-Rad) (for the TGX Stain-Free™ Protein Gel).

### Blotting

The membrane was blocked in Tris Buffered Saline Tween20 (TBST) with 5% skim milk (2 h, 25 °C). The membrane was incubated in blocking buffer (1 h, 25 °C) with Anti-ACE2 Polyclonal Antibody (ab15348, Abcam) at 1:300 dilution, then washed 3–4 times with TBST, and incubated with a goat anti-rabbit Immunoglobulin/HRP secondary antibody (P044801-2, Dako) at 1:5000 in the blocking buffer. The membrane was washed 3–4 times in TBST, then incubated in Clarity Western ECL Substrate (1705060, Bio-Rad) for 5 min and imaged. The density of each band (determined by the ChemiDoc Touch Imaging System) was determined prior to analysis using ImageLab software from Bio-Rad™. Analysis controlled protein loading for each sample by normalizing using stain-free total protein quantification [30] and was further normalized to an internal control sample (pooled TSCs) on each membrane. Samples were run in duplicate and averaged for final analysis.

### Enzyme-linked immunosorbent assay (ELISA)

A commercially available ELISA was used to measure the concentration of ACE (DY929, Duoset R&D Systems) according to the manufacturer’s instructions, using methods previously described [31].

### ACE2 activity assay

ACE2 enzyme activity was assessed according to Xiao and Burns [32] and Tamanna et al. [1]. Briefly, samples (15 µL; cell lysate or cell media) were diluted with enzyme buffer (1 M NaCl, 75 mM Tris-HCl, 5 mM ZnCl_2_; pH 6.5) with protease inhibitors (10 µM captopril, 5 µM amastatin, 10 µM bestatin; Sigma Aldrich, 10 µM Z-prolyl-prolinal; Enzo Life Sciences). MCA-Ala-Pro-Lys(Dnp)-OH (AnaSpec, #AS-60757), an ACE-2 specific fluorescent substrate, was diluted in enzyme buffer and added to samples (final concentration 50 µM). Samples were run in duplicate along with a blank control in a 96-well black microplate. The plate was covered and incubated on a plate shaker (24 h, 25 °C).

The ACE2 standard curve was generated using human recombinant ACE2 protein (R&D Systems, #933-ZN). For each concentration of ACE2 standard, two wells were dedicated to measuring total ACE2 activity and two were dedicated to measuring activity in the presence of DX600 (AnaSpec, #AS-62337), an ACE2 inhibitor. Relative Fluorescence Units (RFU) measured the product MCA-Ala-Pro over 24 h from the substrate MCA-Ala-Pro-Lys(Dnp)-OH as cleaved by ACE2. Fluorescence readings (CLARIOstar Plus, BMG LabTech) were taken with an excitation wavelength of 320 nM, and an emission wavelength of 405 nM.

ACE2 activity was calculated by comparing the RFU of known concentrations of ACE2 standard to the RFU of our samples. As such, ACE2 activity is reported as activity/ng/mL of ACE2.

### Measurement of organoid diameter and assessment of growth

Images of organoid wells were captured using Brightfield microscopy (Olympus Life Science). All wells were imaged in the same orientation. Diameters were measured using QPath Software horizontally across each organoid for *n* = 100 organoids per group, per patient. Symmetrical growth was assessed by comparing the horizontal diameter for each organoid with the vertical diameter for each organoid, for *n* = 100 organoids per group, per patient.

### Statistical analysis

Statistical analysis for differences between groups was undertaken using SPSS Statistics Software. Linear mixed-effects models with random intercepts were used to estimate differences between study groups using restricted maximum likelihood estimation. Differences between groups were considered significant for *p* < 0.05.

All gene variants (i.e. *ACE2*^*+/+*^*,* *ACE2*^*+/*^^*−*^*,* *ACE2*^*−/−*^, *TT*, *CT* and *CC*) were generated for each of the *n* = 3 patient TSC samples, therefore creating an internal control for each patient’s samples within each experiment. All experiments were then replicated three times. As single foetal sex was used, our sample size was chosen to achieve 90% power with an overall alpha level of 5% for nine comparisons, assuming an effect size (f2) of 0.4 and 5 model parameters.

## Results

### *ACE2* knockout (KO) placental organoids

**Fig. 3:**
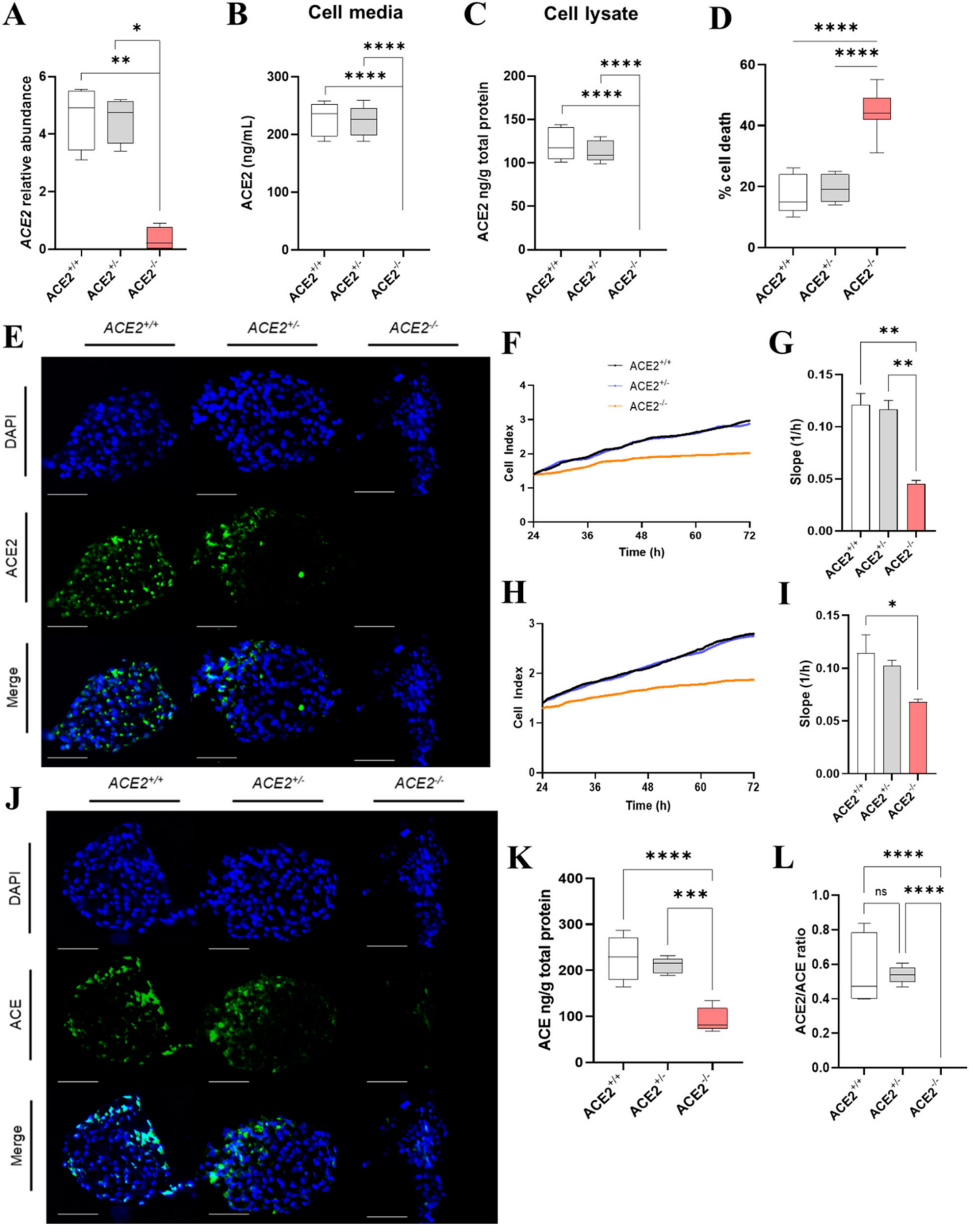
Expression of ACE2 and ACE in *ACE2* KO organoids, and *ACE2* KO cell growth. The abundance of **A** *ACE2* mRNA, **B** ACE2 protein in cell media and **C** ACE2 protein in the cell lysate of *ACE2*^*+/+*^, *ACE2*^*+/-*^ and *ACE2*^*-/-*^ (*ACE2* KO) organoids. **D** Percentage of dead cells in the 24 h post seeding. **E** In situ localisation of ACE2 in *ACE2*^*+/+*^, *ACE2*^*+/-*^ and ACE2 KO organoids. **F** Merged xCELLigence line trajectories for proliferation and **G** proliferation rate (represented by the slope of xCELLigence trajectory, 1/h) of *ACE2*^*+/+*^, *ACE2*^*+/-*^ and *ACE2* KO trophoblast stem cells (TSCs). **H** Merged xCELLigence line trajectories for migration and **I** migration rate of *ACE2*^*+/+*^, *ACE2*^*+/-*^ and *ACE2* KO TSCs. **J** In situ localisation of ACE in *ACE2*^*+/+*^, *ACE2*^*+/-*^ and *ACE2* KO organoids. **K** Level of ACE protein in cell lysate and **L** ACE2/ACE (ACE2:ACE) ratio (from cell lysate levels) of *ACE2*^*+/+*^, *ACE2*^*+/-*^ and *ACE2* KO organoids. Representative immunofluorescence images of organoid sections after 20 days of culture. Scale bars: 100 μm. Nuclei are stained with DAPI. Data are presented as a 10–90 percentile interleaved box-and-whisker plot. Statistics: linear mixed model with random intercept accounting for individual patient correlation. White bars denote *ACE2*^*+/+*^ group, grey bars denote *ACE2*^*+/-*^ group, and red bars denote the *ACE2* KO group. *p < 0.05, **p < 0.01, ***p < 0.001, ****p < 0.0001. ns indicates non-significance (*n* = 9).

*ACE2*^*+/*^^*−*^ *and ACE2*^*−*^^/^^*−*^ (hereafter referred to as ‘*ACE2* KO’) placental organoids were successfully generated. *ACE2*^*+/+*^ *and ACE2*^+/^^*−*^ organoids expressed significantly more *ACE2* mRNA than *ACE2* KO organoids (*p* = 0.00155, *p* = 0.0324 respectively; Fig. 3A). No significant difference in *ACE2* mRNA abundance was detected between *ACE2*^*+/+*^ and *ACE2*^+/^^*−*^ organoids. ACE2 protein was not detected in cell media nor cell lysate of *ACE2* KO organoids (nil detected). Therefore, ACE2 protein was significantly increased in cell media (Fig. 3B) and cell lysate (Fig. 3C, D) of *ACE2*^*+/+*^ and *ACE2*^+/^^*−*^ organoids compared to *ACE2* KO organoids (*p* < 0.0001 for all). No significant difference in ACE2 protein abundance was detected between *ACE2*^*+/+*^ *and ACE2*^+/^^*−*^ organoids in cell media or cell lysate. In situ localisation of ACE2 protein showed that ACE2 was mainly expressed in syncytiotrophoblasts (Fig. 3E). No staining was observed in *ACE2* KO organoids.

### ACE2 knockout leads to slower proliferation and migration, and increased cell death in TSCs

The rate of cell proliferation for *ACE2*^*+/+*^ (slope average 0.121) and *ACE2*^*+/*^^*−*^ (slope average 0.117) TSCs was significantly increased compared with *ACE2* KO TSCs (slope average 0.045; *p* = 0.0015, *p* = 0.0020 respectively; Fig. 3F, G). The rate of cell migration for *ACE2*^*+/+*^ (slope average 0.114) TSCs was significantly increased compared with *ACE2* KO TSCs (slope average 0.068; *p* = 0.0472; Fig. 3H, I) but was not significantly changed when compared with *ACE2*^*+/*^^*−*^ TSCs (slope average 0.103).

*ACE2*^*+/+*^ and *ACE2*^*+/*^^*−*^ TSCs had average cell death proportions of 17% and 19%, respectively. In contrast, *ACE2* KO TSCs had an average cell death proportion of 44%. *ACE2*^*+/+*^ and *ACE2*^*+/*^^*−*^ TSCs had significantly lower proportions of cell death than *ACE2* KO TSCs (*p* < 0.0001 for all, Fig. 3K).

### *ACE2* KO markedly reduces ACE protein expression in placental organoids

In situ localisation of ACE protein showed that ACE was mainly expressed in the outer membrane of organoids (Fig. 3J). *ACE2*^*+/+*^ and *ACE2*^*+/*^^*−*^ organoids expressed significantly more ACE protein than *ACE2* KO organoids (*p* < 0.0001, *p* = 0.0003, respectively; Fig. 3L). Given the absence of ACE2 protein in *ACE2* KO organoids, the ACE2:ACE ratio was significantly higher in *ACE2*^*+/+*^ and *ACE2*^*+/*^^*−*^ organoids compared to *ACE2* KO organoids (*p* < 0.0001 for both; Fig. 3M) but remained unchanged between *ACE2*^*+/+*^ and *ACE2*^*+/*^^*−*^ organoids (no significant difference).

### *ACE2* KO reduces diameter and induces asymmetrical growth in placental organoids

**Fig. 4:**
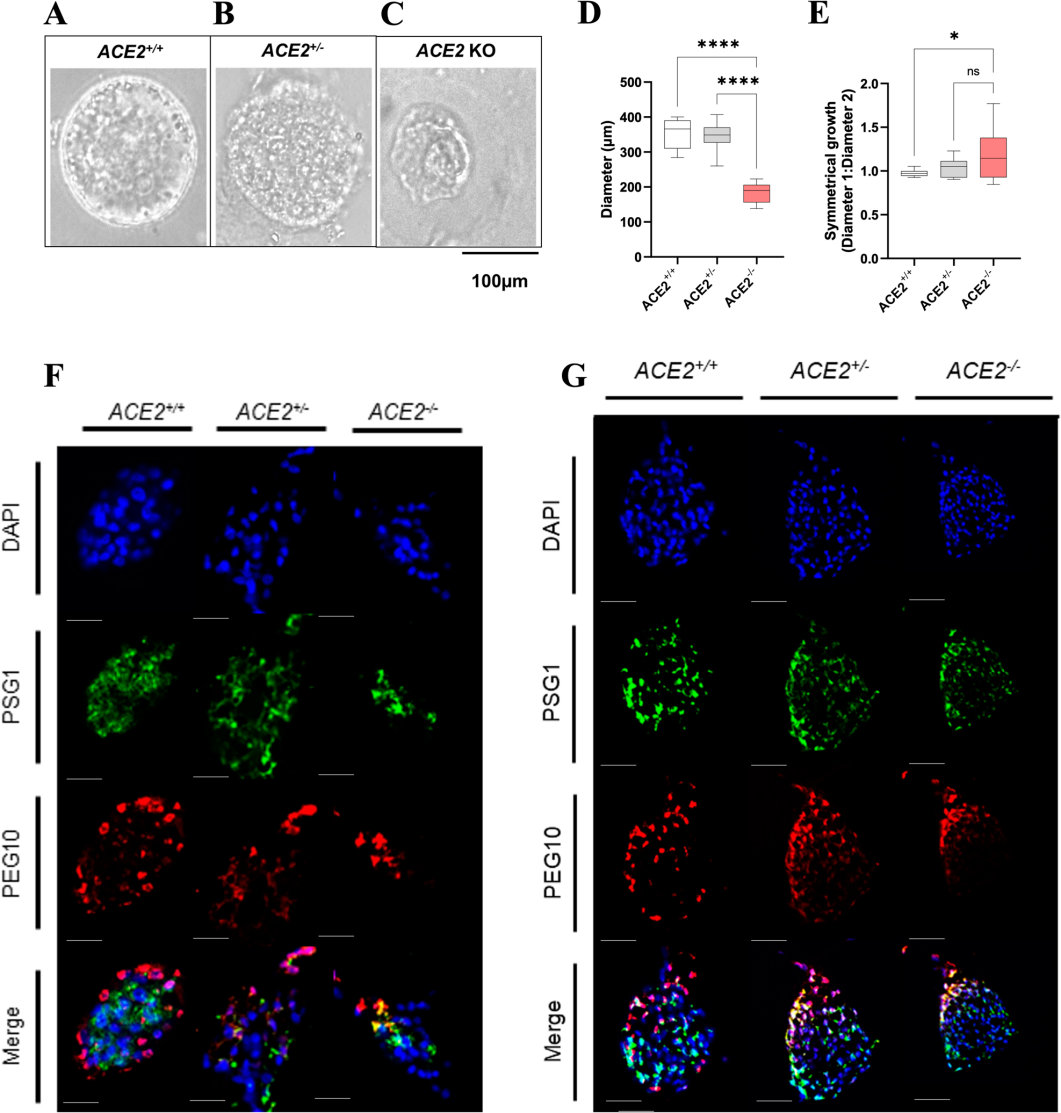
*ACE2* KO organoid growth and trophoblast cell differentiation. Representative image (Brightfield) of symmetrical organoid growth **A** ACE2^+/+^, **B** ACE2^+/^^*−*^ and **C** asymmetrical organoid growth (ACE2 KO). **D** The average diameter (µm) of ACE^+/+^, ACE^+/^^*−*^ and ACE2 KO organoids. **E** The ratio of diameter 1 (horizontal) to diameter 2 (vertical) of ACE^+/+^, ACE^+/^^*−*^ and ACE2 KO organoids as a measure of symmetrical organoid growth. A value of 1.0 indicates perfect symmetry. In situ localisation of PSG1 and PEG10 in **F** early culture (day 6) and **G** late culture (day 28) ACE^+/+^, ACE^+/^^*−*^ and ACE2 KO organoids. Scale bars: 100 µm. PSG1 staining signifies syncytiotrophoblast cells, and PEG10 staining signifies villus cytotrophoblast cells. Nuclei are stained with DAPI. Data are presented as a 10–90 percentile interleaved box-and-whisker plot. Statistics: linear mixed model with random intercept accounting for individual patient correlation. White bars denote the ACE^+/+^ group, grey bars denote the ACE^+/^^*−*^ group, and red bars denote the ACE2 KO group. ns indicates non-significance, *p < 0.05, ****p < 0.0001 (*n* = 9).

Placental organoids tend to exhibit a spherical structure [19, 20]. However, with the *ACE2* knockout, we observed significantly smaller placental organoids with ‘squashed’, i.e. asymmetrical, structures (Fig. 4A–C). We have therefore measured the horizontal and vertical diameters of organoids from each genotype to determine the effect of *ACE2* on growth and symmetry.

*ACE2*^*+/+*^ and *ACE2*^*+/*^^*−*^ organoids had significantly larger diameters compared to *ACE2* KO organoids (*p* < 0.0001 for all, Fig. 4D).

Organoids were significantly less symmetrical in *ACE2* KO organoids compared with *ACE2*^*+/+*^ organoids (*p* = 0.0186, Fig. 4E) only.

All organoids were positively stained for PSG1, which marks syncytiotrophoblast cells, and PEG10, which marks villus cytotrophoblast cells. Given the protocol followed to generate organoids [20], we expected spontaneous syncytialisation of cells on the inside of the organoid, with villus cytotrophoblasts around the outside of the organoid.

In situ localisation of PSG1 protein showed that PSG1 was mainly expressed in cells on the inside of *ACE2*^*+/+*^, *ACE2*^*+/*^^*−*^ and *ACE2* KO organoids both in early (Fig. 4F) and late (Fig. 4G) culture. PEG10 was mainly expressed in cells around the outside of *ACE2*^*+/+*^ organoids but stained asymmetrically in *ACE2*^*+/*^^*−*^ and *ACE2* KO organoids, potentially indicating irregular cell differentiation.

### Placental organoids with *ACE2* rs2074192 induced SNP

**Fig. 5:**
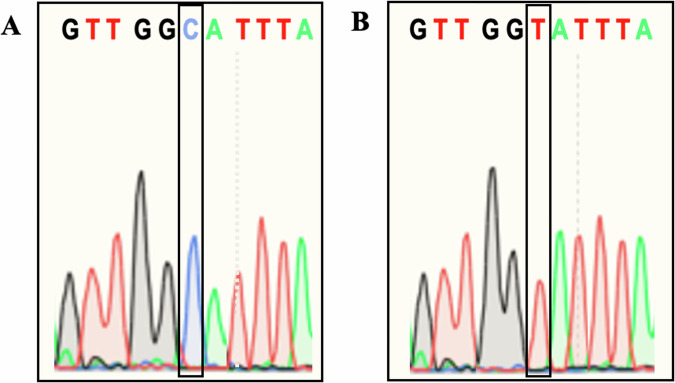
Successful induction of the *ACE2* rs2074192 SNP. Successful gene-editing at position 6: the reference C allele (**A**) and the alternate T allele (**B**).

TSCs were successfully gene-edited to induce the *ACE2* rs2074192 SNP. The plasmid accuracy was confirmed by Sanger sequencing (Fig. 5), which verified a single base substitution from C to T at position 15564667 on the X chromosome (reference GRCh38.p2 38.2/144), *ACE2* gene. Transfected TSCs were genotyped (see methods) to verify successful SNP candidates, from which organoids were created. Three genotype groups were created: homozygous CC, heterozygous SNP (CT) and homozygous SNP (TT).

### Organoids with the *ACE2* rs2074192 TT genotype have increased *ACE2* mRNA and ACE2 protein

**Fig. 6:**
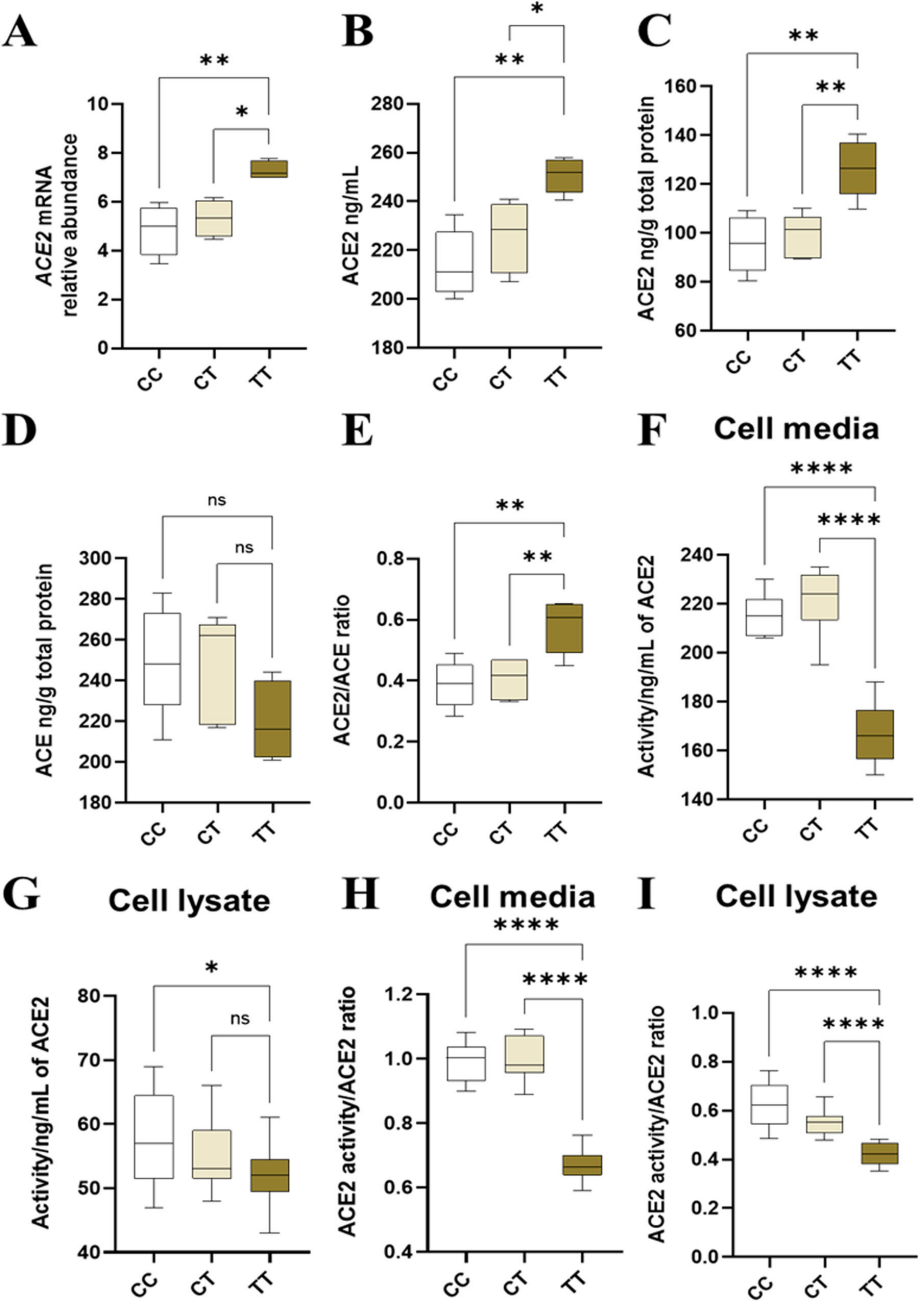
Expression and activity of ACE2 and ACE in CC, CT and TT organoids. The abundance of **A** *ACE2* mRNA, **B** ACE2 protein in cell media and **C** ACE2 protein in cell lysate; **D** Level of ACE protein in cell lysate, **E** ACE2/ACE ratio (from cell lysate levels); enzyme activity of ACE2 in **F** cell media and **G** cell lysate, and the ratio between ACE2 activity and ACE2 levels in **H** cell media and **I** cell lysate of CC, CT and TT organoids. Data are presented as a 10–90 percentile interleaved box-and-whisker plot. Statistics: linear mixed model with random intercept accounting for individual patient correlation. White bars denote the CC group, beige bars denote the CT group, and brown bars denote the TT group. ns indicates non-significance, *p < 0.05, **p < 0.01, ***p < 0.001, ****p < 0.0001 (*n* = 9).

TT organoids had significantly higher expression of *ACE2* mRNA compared with both CC and CT organoids (*p* = 0.0040, *p* = 0.0141, respectively; Fig. 6A). TT organoids also had significantly higher levels of ACE2 protein than CC and CT organoids in both the cell media (*p* = 0.0013, *p* = 0.0169, respectively; Fig. 6B) and cell lysate (*p* = 0.0018, *p* = 0.0040, respectively; Fig. 6C, D). There was no significant difference in the abundance of *ACE2* mRNA, nor ACE2 protein in cell media or lysate, between CC and CT organoids.

Time to confluence, and percentage of cell death, were also assessed, but no significant differences were detected between groups (Supplementary Fig. 2).

### *ACE2* rs2074192 genotype did not change ACE protein expression, but increased the ACE2/ACE ratio in TT organoids

There was no significant difference in ACE protein level (measured in cell lysate) between CC, CT and TT organoids (Fig. 6E). However, the ACE2/ACE ratio was significantly higher in TT organoids compared to both CC and CT organoids (*p* = 0.0049, *p* = 0.0093, respectively; Fig. 6F). There was no significant difference in ACE protein expression, nor ACE2/ACE ratio, between CC and CT organoids.

### ACE2 activity is decreased in *ACE2* rs2074192 TT organoids

ACE2 enzyme activity was significantly reduced in the cell media of TT organoids compared to both CC and CT organoids (*p* < 0.0001 for both; Fig. 6G). There was no significant difference in ACE2 activity in the cell media of CT organoids compared to CC organoids. ACE2 enzyme activity was significantly reduced in the cell lysate of TT organoids compared to CC organoids (*p* = 0.0303; Fig. 6H). There was no significant difference in ACE2 activity in the cell lysate of CT organoids compared to TT organoids, nor compared to CC organoids.

The ratio between ACE2 activity and ACE2 levels was significantly decreased in the cell media of TT organoids compared to both CC and CT organoids (*p* < 0.0001 for both; Fig. 6I), while there was no significant difference between CT and CC organoids. The ratio between ACE2 activity and ACE2 levels was significantly decreased in the cell lysate of TT organoids compared to both CC and CT organoids (*p* < 0.0001 for both; Fig. 6J), while there was no significant difference in the ratio between CT and CC organoids.

### *ACE2* rs2074192 TT organoids have reduced diameters and asymmetrical growth

**Fig. 7:**
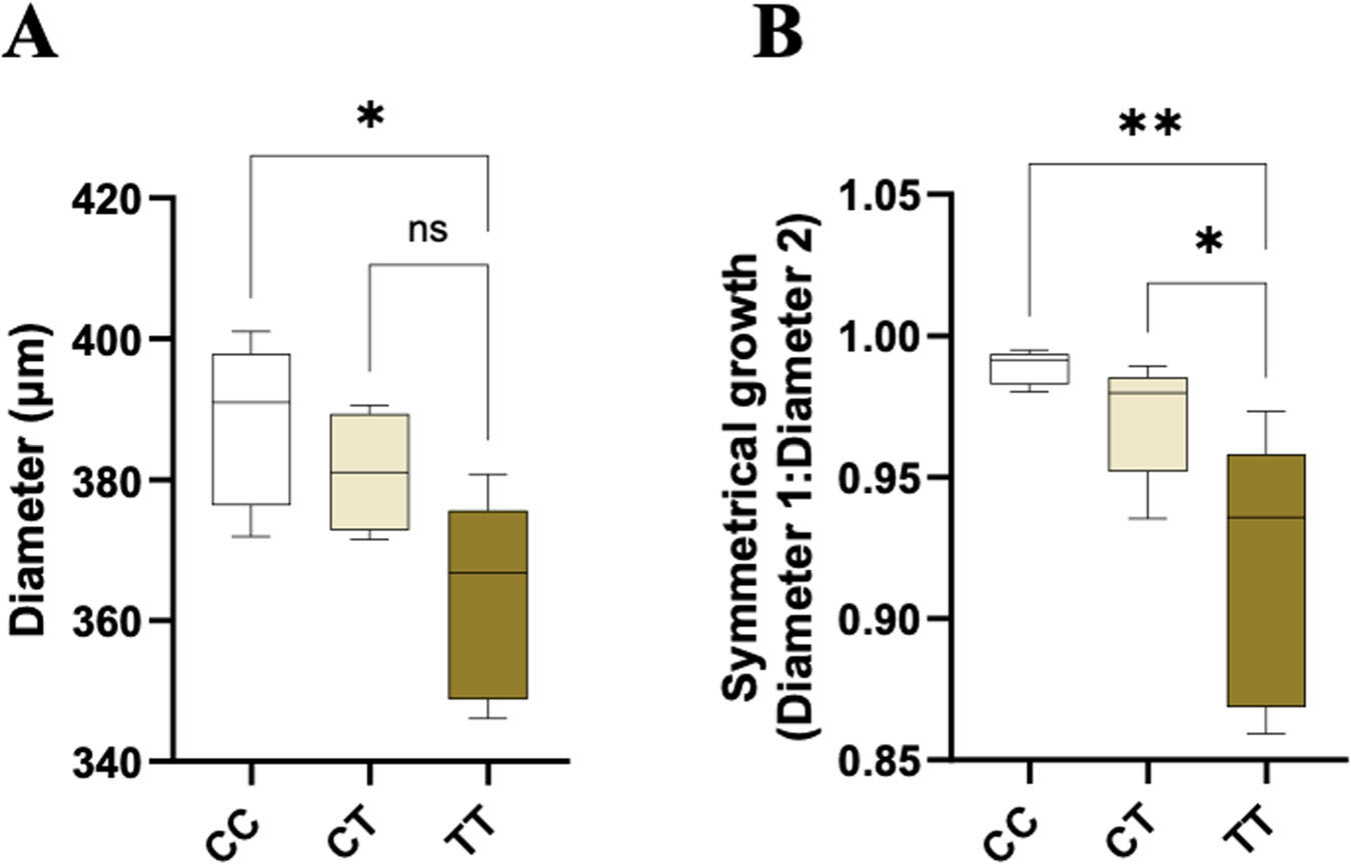
Asymmetrical growth of TT organoids. **A** The average diameter (µm) of CC, CT and TT organoids. **B** The ratio of diameter 1 (horizontal) to diameter 2 (vertical) of CC, CT, and TT organoids as a measure of symmetrical organoid growth. A value of 1.0 indicates perfect symmetry. Data are presented as a 10–90 percentile interleaved box-and-whisker plot. Statistics: linear mixed model with random intercept accounting for individual patient correlation. White bars denote the CC group, beige bars denote the CT group, and brown bars denote the TT group. ns indicates non-significance, *p **<** 0.05, **p **<** 0.01 (*n* **=** 9).

The diameter of TT organoids was significantly reduced compared to CC organoids (*p* = 0.0141; Fig. 7A) only. Organoid growth was significantly less symmetrical in TT organoids compared to both CC and CT organoids (*p* = 0.0077, *p* = 0.0415, respectively; Fig. 7B). There was no significant difference in diameter or symmetrical growth measurements between CC and CT organoids.

## Discussion

This study is the first to successfully use gene-editing to stably produce *ACE2* knockout placental organoids, and the first to induce a SNP in placental organoids. Using these models, the study demonstrates that *ACE2* is important for cell growth (as evidenced by changes in the time to confluence and percentage cell death of TSCs), as well as placental organoid size and growth (as evidenced by changes in organoid diameters and symmetry growth measurements). Furthermore, we show that the ACE2:ACE ratio is disrupted in both organoid models, resulting in impaired growth and development. Our data are consistent with our hypothesis that *ACE2* plays an important role in placental development, and that ACE2 and ACE need to exist in a finely tuned balance to ensure proper growth and morphogenesis. We have also confirmed that the *ACE2* rs2074192 TT genotype results in increased ACE2 mRNA and protein, and altered ACE2:ACE ratios, which likely mediate the effects on TSC and organoid growth and cell survival.

*ACE2* knockout mouse models of pregnancy have been useful in reinforcing the importance of ACE2 in placentation and pregnancy health. This study has extended our understanding of the role of ACE2 in the placenta, creating the first *ACE2* knockout in primary human placenta cells. Furthermore, we have explored the molecular implications of the carriage of a common *ACE2* SNP, rs2074192. *ACE2* variants are associated with pregnancy complications, as well as with a number of other diseases characterised by renin-angiotensin system dysfunction, including COVID-19, hypertension [14, 33] and heart failure [17]. *ACE2* rs2074192 is a common SNP, with an estimated frequency of C (reference allele) = 0.63 and T (alternate allele) = 0.37, according to a study of 102,615 people (including varied ethnicities) in the genome aggregation database [18]. This suggests that at least one copy of this SNP naturally occurs in over one-third of the population, and a recent study indicated that the incidence of the TT genotype in their study population was 19.8% [10]. This highlights the importance of understanding the mechanism by which carriage of the alternate allele increases the risk of disease.

In silico analysis of *ACE2* rs2074192 suggests that it increases splicing donor sites, creating a changed secondary RNA structure, and affecting protein production and activity. As *ACE2* rs2074192 is an intronic polymorphism, it is likely that it indirectly positively regulates gene expression by altering ACE2 splicing and transcription enhancement [15]. This study confirms that carriage of the TT genotype, but not the CT genotype, results in increased placental ACE2 mRNA and protein expression. This has major implications for the pathogenesis of diseases dependent on ACE2 expression, including pregnancy complications such as preeclampsia [1] and SGA [1], and infection with SARS-CoV-2 virus [34], which can directly infect syncytiotrophoblasts through earlier endometrial cell infection [35]. However, the mechanism of action by which this SNP contributes to pathogenesis requires further study. Both the *ACE2* rs2074192 (C to T) SNP and haplotypes CAGC and TAGT are associated with altered levels of the Ang 1–7 peptide, a direct product of ACE2 activity [10]. As this study did not quantify Ang 1–7 expression, further studies should investigate whether Ang 1–7 is altered as a result of *ACE2* rs2074192 genotype, providing additional information on a potential mechanism for pathogenesis of diseases dependent on ACE2 in T allele carriers.

This study observed that *ACE2* knockout TSCs took longer to reach confluence and had higher percentages of cell death in culture. This finding has implications for pregnancy complications with origins in early gestation, when TSCs play an important role in placental development that underpins subsequent function. During early gestation, the placenta undergoes rapid trophoblast proliferation and differentiation [36]. Not only did this study observe slower TSC growth and higher levels of cell death in *ACE2* knockouts, but *ACE2* knockout organoids had smaller diameters, as well as asymmetrical growth, reflecting impaired organoid development. Interestingly, although no change in TSC growth or cell death was observed with the *ACE2* rs2074192 genotype, TT organoids also had smaller diameters and asymmetrical growth.

This impaired growth has concerning implications for placental development. Human placental organoids provide an innovative model for studying placental development in vitro. Ethical limitations to the study of early- to mid-gestation placentae, created the need for physiologically relevant human placental models with 3D architecture. Current models are limited [37–39] while mouse placenta differs both in structure and endocrine function [40]. Organoids recapitulate all first-trimester placental functions, can differentiate into the expected cell lineages, and are able to be cultured long-term without the loss of these features [41]. For this reason, human placental organoids are the closest possible parallel to studying the early gestation human placenta in vivo. *ACE2* gene-editing of placental organoids in this study provides insight into the detrimental effect of *ACE2* knockout and rs2074192 TT genotype on placental development, likely leading to aberrant placentation in human pregnancy.

Whilst an absence of ACE2 is clearly detrimental to cell growth and increases cell death, a complete knockout is highly unlikely in nature and thus is less biologically relevant for causal inference. Therefore, we have compared the knockout and induced SNP models to make inferences most biologically relevant to human pregnancy. Surprisingly, both the *ACE2* KO organoids and the induced SNP organoids exhibited diminished growth and symmetry, despite *ACE2* KO organoids having markedly reduced ACE2 protein, and induced SNP organoids having markedly increased protein, compared to their respective controls. This result suggests that the absolute expression value of ACE2 is the not sole determinant of organoid growth.

One common feature between *ACE2* knockout organoids and TT organoids is the altered ACE2:ACE ratio. Our data suggest that ACE2 and ACE, which are known to partly control cell growth [3], have more contributions to placental organoid development (as evidenced by organoid symmetry and growth) than simply by affecting cell proliferation and death. This is evident from our observation of unchanged TSC growth and cell death levels in TT TSCs, despite them also having altered ACE2 and ACE expression.

The mechanism for this unexpected increase in ACE2 protein, but decreased organoid growth and symmetry, could be due to the reduced ACE2 enzyme activity observed in TT organoids. *ACE2* rs2074192 is an intronic SNP, which does not directly impact protein-coding regions of the genome. Therefore, the contribution of this SNP to ACE2 protein increase is indirect, likely through a complex mechanism not yet elucidated. We hypothesise that the excess of ACE2 protein in TT organoids has resulted in a compensatory response in the cell, reducing the *activity* of ACE2 in an attempt to re-establish an ACE2:ACE equilibrium. This theory is supported by the literature, showing that *ACE2* rs2074192 is associated with an increased risk of SGA [5], a condition in which inadequate placentation often leads to smaller placentae and a smaller foetus. Furthermore, in SGA, ACE2 activity is significantly reduced [1].

This study indicates that, whilst ACE2 expression is significant, the activity of the enzyme and maintenance of the ACE2 and ACE ratio in equilibrium (in this paper termed an ACE2:ACE ratio) may be of greater importance. Studies investigating pregnancy complications support this conclusion. In preeclampsia, a potentially life-threatening complication characterised by maternal hypertension and multi-organ failure, ACE2 activity reflected by the ACE2:ACE ratio is higher than normal, impairing placental development and function [1]. In SGA, placental expression of ACE and ACE2 at delivery are both higher compared with uncomplicated pregnancy. Birthweight centiles are negatively correlated with maternal plasma ACE and ACE2 levels in mid-pregnancy, suggesting that tight regulation of these enzymes is critical to ensure pregnancy health [1]. As previously mentioned, the ratio of ACE2 activity to ACE2 levels in maternal plasma is significantly reduced in SGA, indicating that ACE2 operates with reduced efficiency in this condition [1]. This could be due to the increased presence of an endogenous inhibitor [42] in SGA, although this has never been investigated. Placental ACE2 deficiency, and the associated elevation in placental Ang II levels, negatively impact pregnancy by impairing maternal weight gain and restricting foetal growth [2]. This implies the balance of ACE2:ACE supersedes the importance of ACE2 levels in isolation in determining foetal growth. Indeed, herein we observed similar reductions in organoid diameter and growth between the complete *ACE2* knockout and rs2074192 models, despite the difference in absolute ACE2 expression values.

Interestingly, this study showed that *ACE2* rs2074192 TT organoids not only had elevated ACE2 protein within cells (as assessed by cell lysate protein levels) but also secreted more ACE2 protein into the cell media. Whilst ACE2 is typically a membrane-bound peptidase, it can also be shed from the membrane [43], producing soluble ACE2 (sACE2). It should be noted that this study utilised only an ACE2 antibody specific to the membrane-bound ACE2 protein and we cannot comment on the production of sACE2 by these organoid models. Future studies will utilise a specific sACE2 antibody to examine this.

A limitation of this study is that it only utilised placental tissue from female fetuses. This is because placental expression of ACE and ACE2 is foetal sex-dependent. Both ACE and ACE2 are more highly expressed in placentae from females compared to male fetuses [44]. For ACE2, this is likely due to the fact that *ACE2*, located on the X-chromosome, escapes X chromosome inactivation, thus often two copies are expressed instead of one in females [45]. The balance of downstream effector peptides Ang II (via ACE) and Ang 1–7 (via ACE2) is also influenced by foetal sex. In early gestation, Ang 1–7:Ang II ratios are reduced in the placenta of females, indicating less Ang 1–7 production as a result of reduced ACE2 activity [46]. Female babies are on average smaller and more likely to be growth-restricted than males [47, 48]. In this study, the organoid growth symmetry only significantly changed between the *ACE2*^*+/+*^ and total knockout, but not *ACE2*^*+/*^^*−*^ and the knockout. This could indicate that in males, where only one *ACE2* gene copy is present (due to the presence of only one X chromosome), there may be differences in placental development. Thus, *ACE2* foetal sex-specific molecular roles require further investigation. Future studies should include male samples, as well as females to expand on the findings from this study.

Our data clearly demonstrate that ACE2 plays an important role in placental organoid development, and its expression is increased with the rs2074192 TT genotype. Importantly, ACE2 enzyme activity and the ACE2:ACE ratio appeared to make more difference to the growth and development of TSCs and placental organoids than the absolute expression values of ACE2 alone. We anticipate that this study will be used to facilitate a deeper understanding of the role of ACE2 in placental development. The gene-editing technique in placental organoids that we have developed could be used to study the roles of many other genes and their variants on placental growth and function.

## Supplementary information

Supplementary Figure Legends

Supplementary Figure 1

Supplementary Figure 2

## Data Availability

The authors declare that the data supporting the findings of this study are available within the paper and its Supplementary Information files. Should any raw data files be needed in another format they are available from the corresponding author upon reasonable request.
