# Supplementary material for: Genetically edited human placental organoids cast new light on the role of ACE2
Source: Cell Death Dis. 2025 Feb 7;16(1):78. doi: 10.1038/s41419-025-07400-x (PMC11806113; doi:10.1038/s41419-025-07400-x)
Supplement: Supplementary file 1 — Supplementary Figure Legends [file 41419_2025_7400_MOESM1_ESM.docx]

***Supplementary Figure 1.*** *Representative full-length immuno blot membrane images showing ACE2 protein expression in* ***(A)*** *ACE2^+/+^ (++), ACE2^+/-^ (+-) and ACE2^-/-^ (--) organoids and* ***(B)*** *CC, CT and TT organoids. Arrow indicates 117 kDa, predicted molecular weight.*

Supplementary Figure 2. (A) Percentage of cells that died in 24 hours post-seeding, and (B) number of hours post-seeding for cells to reach confluence of CC, CT and TT TSCs. Data are presented as a 10-90 percentile interleaved box-and-whisker plot. Statistics: linear mixed model with random intercept accounting for individual patient correlation. White bars denote CC group, beige bars denote CT group, brown bars denote TT group. ns indicates non-significance. (n=9)
