# Supplementary figures and images for: Genetically edited human placental organoids cast new light on the role of ACE2
Source: Cell Death Dis. 2025 Feb 7;16(1):78. doi: 10.1038/s41419-025-07400-x (PMC11806113; doi:10.1038/s41419-025-07400-x)

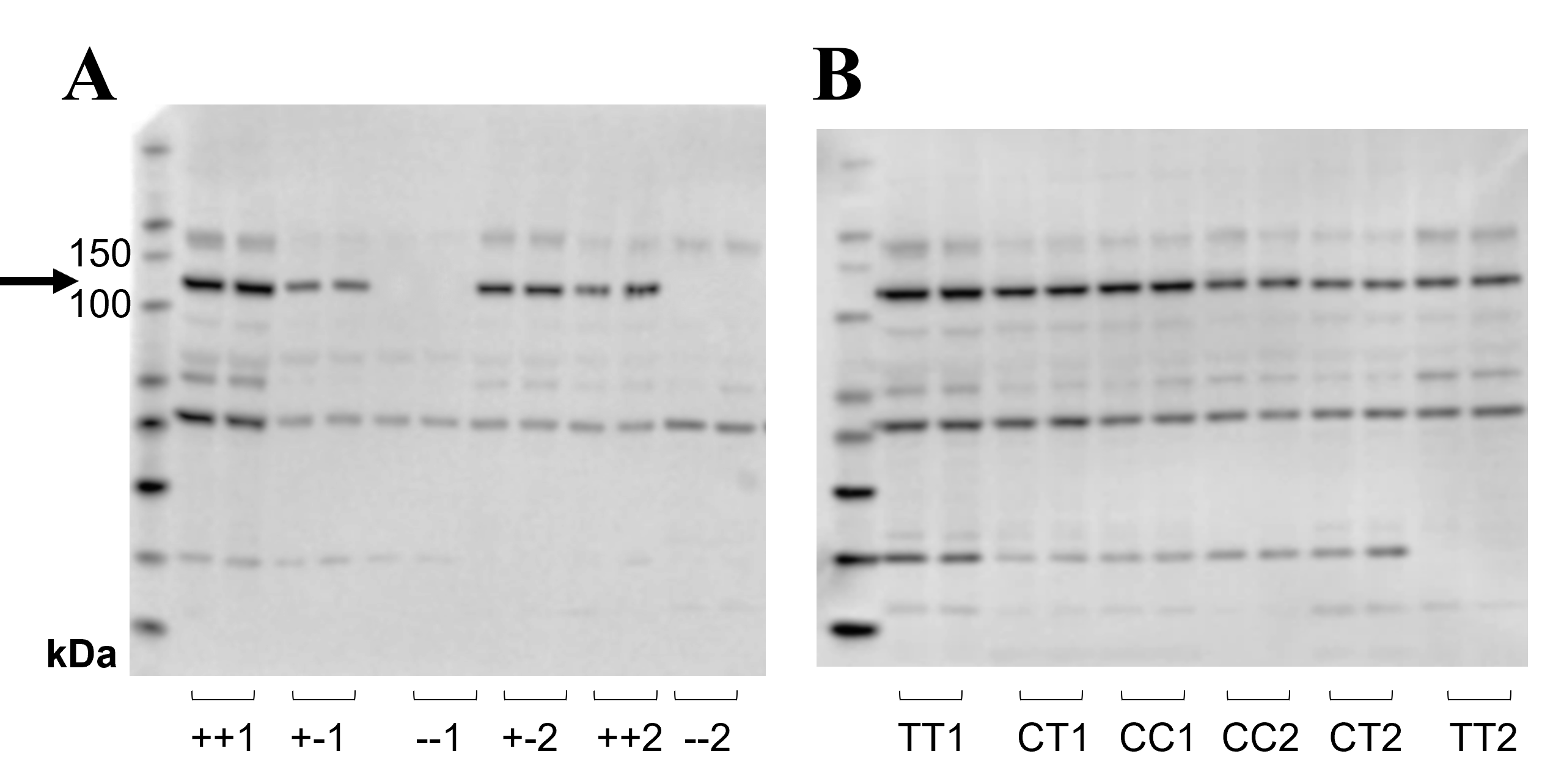

Supplement: Supplementary file 2 — Supplementary Figure 1 [file 41419_2025_7400_MOESM2_ESM.png]

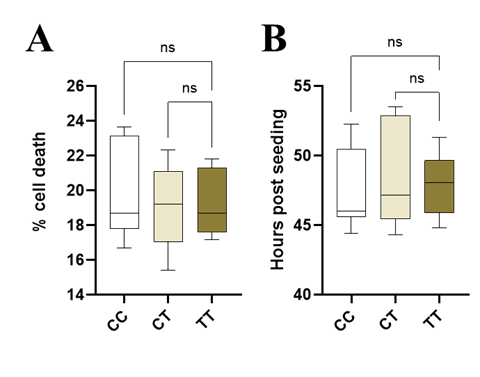

Supplement: Supplementary file 3 — Supplementary Figure 2 [file 41419_2025_7400_MOESM3_ESM.png]
